# Cyclodextrins, Natural Compounds, and Plant Bioactives—A Nutritional Perspective

**DOI:** 10.3390/biom11030401

**Published:** 2021-03-09

**Authors:** Svenja Wüpper, Kai Lüersen, Gerald Rimbach

**Affiliations:** Institute of Human Nutrition and Food Science, University of Kiel, Hermann-Rodewald-Strasse 6, 24118 Kiel, Germany; luersen@foodsci.uni-kiel.de (K.L.); rimbach@foodsci.uni-kiel.de (G.R.)

**Keywords:** cyclodextrins, gamma-cyclodextrin, complex, ursolic acid, oleanolic acid, betulinic acid, propolis, tocotrienol, curcumin

## Abstract

Cyclodextrins (CDs) are a group of cyclic oligosaccharides produced from starch or starch derivatives. They contain six (αCD), seven (βCD), eight (γCD), or more glucopyranose monomers linked via α-1,4-glycosidic bonds. CDs have a truncated cone shape with a hydrophilic outer wall and a less hydrophilic inner wall, the latter forming a more apolar internal cavity. Because of this special architecture, CDs are soluble in water and can simultaneously host lipophilic guest molecules. The major advantage of inclusion into CDs is increased aqueous solubility of such lipophilic substances. Accordingly, we present studies where the complexation of natural compounds such as propolis and dietary plant bioactives (e.g., tocotrienol, pentacyclic triterpenoids, curcumin) with γCD resulted in improved stability, bioavailability, and bioactivity in various laboratory model organisms and in humans. We also address safety aspects that may arise from increased bioavailability of plant extracts or natural compounds owing to CD complexation. When orally administered, α- and βCD—which are inert to intestinal digestion—are fermented by the human intestinal flora, while γCD is almost completely degraded to glucose units by α-amylase. Hence, recent reports indicate that empty γCD supplementation exhibits metabolic activity on its own, which may provide opportunities for new applications.

## 1. Introduction

**Figure 1:**
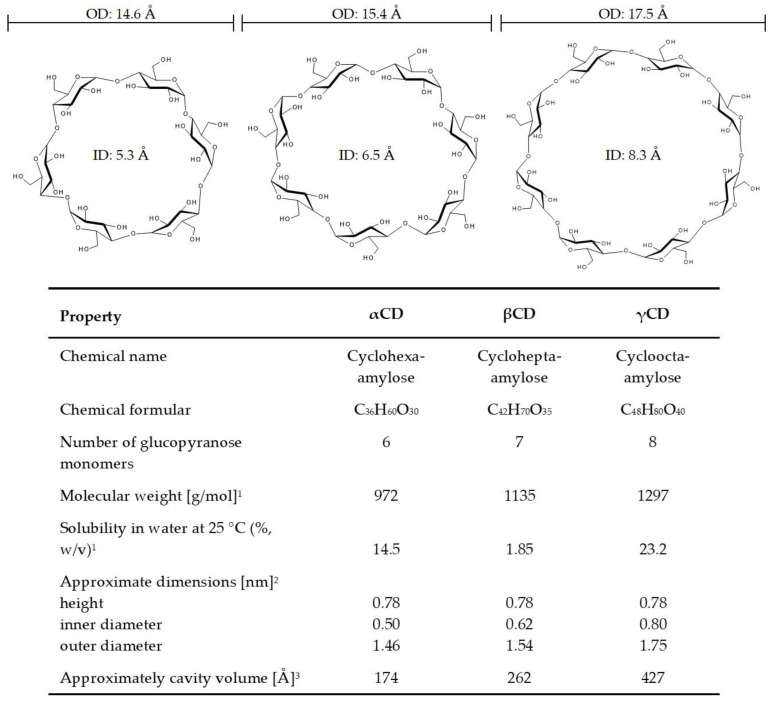
Chemical structures and physicochemical properties of the three main cyclodextrins α-, β-, and γ-cyclodextrin (from left to right αCD, βCD, and γCD). ID: Inner diameter; OD: Outer diameter. ^1^ From [2]. ^2^ From [5]. ^3^ From [1].

Cyclodextrins (CDs), also known as cyclomaltoses, cycloamyloses, and Schardinger dextrins, are a group of cyclic oligosaccharides that are produced from starch or starch derivatives by the bacterial enzyme cyclodextrin glycosyltransferase (CGTase) [1,2]. CDs contain six or more glucopyranose monomers linked via α-1,4-glycosidic bonds. The most prominent members of the CD family are α- (six glucopyranose units), β- (seven glucopyranose units), and γCD (eight glucopyranose units, Figure 1) [1]. Through the specific steric arrangement of their glucose units, CDs are soluble in water and can function as important complexation agents for a broad range of more lipophilic molecules, including natural compounds and plant bioactives, with increasing use in industrial and research applications [1,3]. The major advantage of the inclusion of lipophilic substances into CDs is increased aqueous solubility with enhanced stability and bioavailability of these guest molecules. In addition, CDs can be used to prevent drug-drug and drug-excipient interactions, convert liquid drugs into microcrystalline powder, and reduce gastrointestinal drug irritation [4].

This review aims to provide a short historical overview as well as a summary of the synthesis, properties, pharmacokinetics, and safety of CDs. Furthermore, the differences among the three main CD types are addressed with respect to their complex formation and route of administration. The present state of knowledge regarding the stability, bioavailability, and bioactivity of γCD complexes with selected natural compounds and dietary plant bioactives is described. Finally, the biological activity of empty γCD is also reviewed.

## 2. History of Cyclodextrins

**Figure 2:**
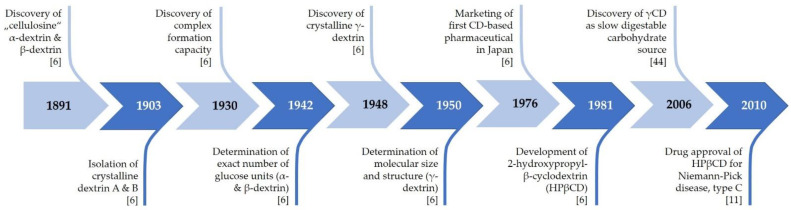
Milestones of cyclodextrin research history. CD, cyclodextrin; γCD, gamma-cyclodextrin; HPβCD, 2-hydroxypropyl-beta-cyclodextrin.

α- and βCD were discovered in 1891 by the French pharmacist and chemist Antoine Villiers as digestion products of potato starch obtained by using the bacterium *Bacillus amylobacter* (Figure 2). Villiers named the novel carbohydrates “cellulosines” α- and β-dextrin and described them as crystalline and slightly sweet [6]. At the beginning of the 20th century, the Austrian microbiologist Franz Schardinger, later known as the “founding father” of CD chemistry, isolated CDs, which he termed “crystalline dextrins”, from several sources of starch following digestion by *Bacillus macerans*. Notably, *B. macerans* is still the most commonly used bacterial species for the production of CGTase to form CDs [2,6,7]. In the 1940s, the German chemist Freudenberg and his coworkers discovered γCD and subsequently solved the cyclic oligosaccharide structure of CD molecules. After discovering the feasibility of preparing CD-inclusion complexes, Freudenberg, Cramer, and Plieninger obtained the first CD-related patent in 1953, which marks the starting point for the application of CDs in drug formulations [8], so that they were no longer just part of academic research, but also part of industrial applications. In 1957, the American chemist and biochemist Dexter French reported on two larger CDs, δ-dextrin and ε-dextrin, with 9 and 10 glucose monomers, respectively [9,10]. However, compared to those of the smaller CDs, the physicochemical properties of these larger CDs were found to be less suitable for complex formation [8]. In 1980, Saenger found that the amylase CGTase is responsible for the conversion of starch or starch derivatives into CDs via a cyclization reaction. Most importantly, this discovery enabled the mass production of CDs [2,7].

Roughly, the history of CDs can be classified into two main research fields. On the one hand, a main focus is the complexation property of CDs and their role as host molecules to improve the properties of guest molecules. As mentioned above, this traditional research area started in the 1950s, has become well established and is still continuing. With the turn of the new millennium, a new branch of CD research came to the fore, after the realization that empty CDs exhibit bioactivity on their own and have potential health benefits. Hence, CD molecules per se have received attention in research, and this topic is increasingly recognized as important [6,11].

## 3. Synthesis of Cyclodextrins

**Figure 3:**
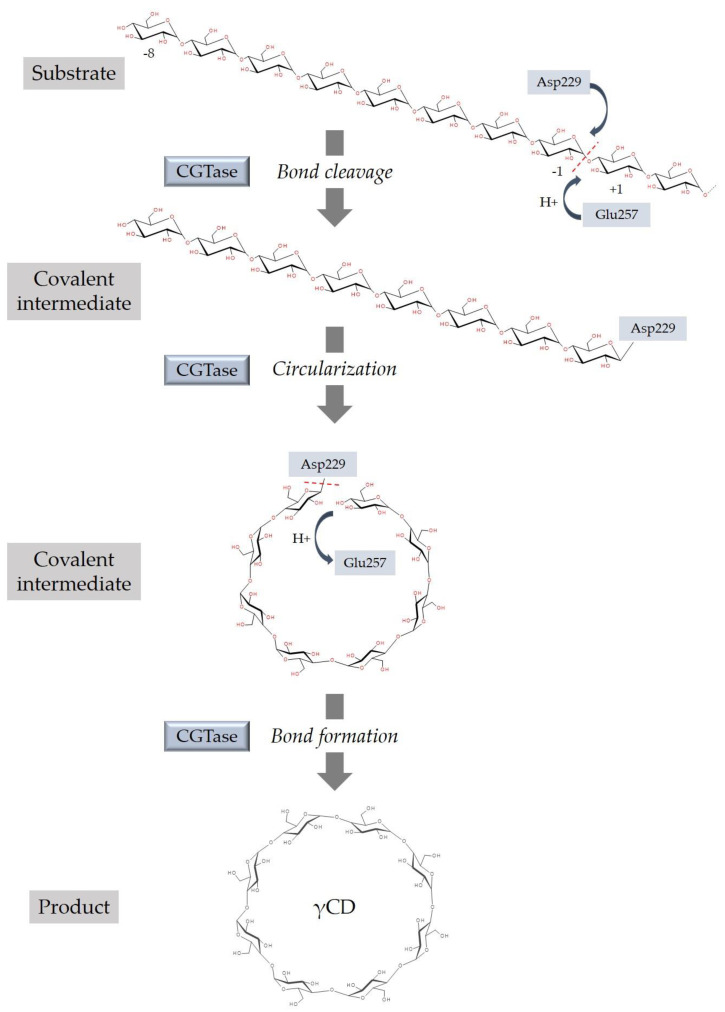
Schematic illustration of γ-cyclodextrin synthesis. The catalyzing enzyme CGTase (1,4-α-D-glucan 4-α-D-(1,4-α-D-glucano)-transferase) first cleaves an α-glycosidic bond in starch by a nucleophilic attack of the amino acid residue Asp229. The generated linear malto-oligosaccharide intermediate is covalently β-1,4-linked to Asp229. In a second step, the oligosaccharide-enzyme intermediate is cleaved under the formation of a cyclic oligosaccharide molecule by closing an α-1,4-glycosidic bond between the two outer glucose units. In the cleavage step, the amino acid residue Glu257 functions as a proton donor for the subsite +1 glucose unit of the leaving starch molecule and, subsequently, in the cycling step as a proton acceptor. Adapted from [1,14]. γCD, gamma-cyclodextrin.

The amylase CGTase (1,4-α-D-glucan 4-α-D-(1,4-α-D-glucano)-transferase) is a unique extracellular enzyme capable of catalyzing the cyclization reaction to form nonreducing CDs from starch, amylose, and other polysaccharides. In addition, CGTase catalyzes two other main transglycosylation reactions (coupling, disproportionation) and exhibits a weak hydrolyzing activity. A common feature of the reactions catalyzed by CGTase is the cleavage of an α-glycosidic bond. However, coupling is the reverse reaction of cyclization, where CD is the substrate and the generated linear malto-oligosaccharide is transferred to an acceptor substrate. During disproportionation, a linear malto-oligosaccharide is cleaved, and the shorter product is transferred to an acceptor substrate [1,12,13]. The γ-cyclization reaction starts with the bond cleavage of polysaccharides. As a result, a covalent intermediate is formed. The two ends of the linear chain of the intermediate will be joined to form a cyclic oligosaccharide (Figure 3) [1,5]. The most crucial catalytic amino acid residues implicated in bond cleavage are Asp229 and Glu257 [1]. During bond formation, an α-1,4-glycosidic bond is closed again [1].

CGTases are found in many bacteria, but have also been identified in archaea. Mainly for historical reasons, CGTases from *Bacillus* species are the most extensively studied enzymes [13]. All known CGTases convert starch to a mixture of α-, β-, and γCDs [12]. Depending on the enzyme source, the product mixtures differ in their relative amounts of α-, β-, and γ-CDs. Hence, CGTases can be further classified according to their major CD products [5]. To obtain individual α- and γCDs from the produced mixture, a costly and time-consuming separation is needed [1]. Therefore, CGTases producing a high amount of a single-type CD are often used and, in addition, have been genetically improved [1,13]. For example, *Bacillus thuringiensis* strain GU-2 was isolated from soil as a specific γCD-producing strain with a purity of >95% when starch was used as the substrate. The optimum conditions were reported to be pH 8.5 and 37 °C [15]. Compared to α- and γCD, the separation and purification of βCD is relatively easy, and therefore rather inexpensive due to its low solubility in water [13].

## 4. Properties of Cyclodextrins

The CDs of the so-called “first generation” or “parent cyclodextrins” are α-, β-, and γCD. These polysaccharides consist of six, seven, or eight glucopyranose monomers linked via α-1,4-glycosidic bonds (Figure 1) [2], forming a truncated cone shape with a central axial cavity [16]. Main properties of the three CDs are listed in Figure 1.

The CD molecules have a hydrophilic outer wall because of the free hydroxyl groups on the outside of the molecules and a less hydrophilic or hydrophobic inner wall imparted by the ether oxygen atoms in the glycosidic hemiacetals and the carbon-hydrogen atoms [11]. Accordingly, CDs are soluble in water, yet can form complexes with many hydrophobic guest molecules [2] through the slightly apolar internal cavities. Binding studies by Heredia et al. [17] and Kajtár et al. [18] indicated that the internal polarity of both β- and γCD are comparable with alcoholic solutions. Moreover, as shown by Street and Acree [19], the estimated dielectric constants for the internal cavities of β- and γCD are only slightly different. Since the depth of the cavities is similar in all parent CDs, their inner diameter, which increases with the number of glucose subunits and determines the cavity size (Figure 1), represents the main limiting factor for the ability to host organic molecules. In good accordance to the increasing cavity size, αCD can harbor up to 6.6 molecules water/molecule, βCD up to 11 molecules water/molecule, and γCD up to 17 molecules water/molecule [16].

**Figure 4:**
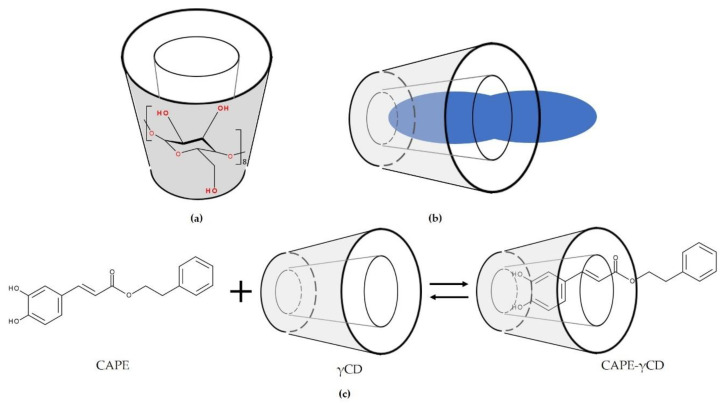
Conical shape of cyclodextrins and complex formation. (**a**) The secondary hydroxyl groups of the glucose molecules are located at the broadest end of the toroid, while the primary hydroxyl groups constitute the narrowest end of the γ-cyclodextrin toroid, here shown representatively for cyclodextrins. (**b**) Schematic illustration of cyclodextrin and guest molecule forming a complex in a 1:1 molar ratio. Adapted from [25]. (**c**) Chemical structure of caffeic acid phenethyl ester (CAPE) and illustration of complex formation of CAPE and γ-cyclodextrin. Adapted from [23,26]. CAPE, caffeic acid phenethyl ester; CAPE-γ-cyclodextrin (CAPE-γCD); γCD, γ-cyclodextrin.

In addition to the central cavity, an essential feature of CDs is the location of the hydroxyl groups [20]. There are secondary hydroxyl groups at the broadest end of the toroid bonded to glucose units at C2 and C3 atoms (Figure 4a). At the opposite end, primary hydroxyl groups are attached to glucose at C6 atoms. These hydroxymethyl groups define the narrowest end of the CD molecule with the smallest cavity diameter. This is based on the property of free rotation from hydroxymethyl groups [21]. Advantages from complexation with CDs are higher water solubility, stability, diffusibility, and bioavailability of guest molecules [22], and masking of ill smell and taste of guest molecules [3]. For example, a propolis-γCD complex possesses a less pungent taste, is water soluble, and stable to heat compared to propolis [23]. To further enhance the water solubility as well as the ability to form inclusion complexes with parent CDs, changes in their free hydroxyl groups have been made [24], resulting in CD derivatives such as 2-hydroxypropyl-γCD (HPγCD).

The capacity to form inclusion complexes with lipophilic substances resulted in a broad range of applications for CDs, especially in analytical chemistry, agriculture, the pharmaceutical field, food, and hygienic articles [3]. When describing CDs as tasteless, odorless, nondigestible, noncaloric, and noncariogenic for humans [27], one should keep in mind that the three CD molecules have somewhat different properties. The native CDs are fermented by the human intestinal flora [21]. However, γCD differs from the other two parent CDs in that it is degraded by amylases to form glucose and other low molecular weight sugar molecules [5,22], which is important for oral administration and thereby calls into question the nondigestible and noncaloric specifications. In turn, undigested γCD is available for fermentation. The metabolic fate of α- and βCD is quite similar to that of other nondigestible but fermentable carbohydrates [27], thereby providing energy for colonic epithelial cells, mainly in the form of butyrate [28]. Furthermore, γCD shows the highest water solubility [5,22]. In addition, γCD has a great advantage over αCD and βCD in trapping larger molecules because it has the largest internal cavity volume (Figure 1) [1].

## 5. Absorption, Distribution, Metabolism, and Excretion of Cyclodextrins

Based on pharmacokinetic studies of CDs, orally administered CDs have low bioavailability, and systemically absorbed CDs disappear rapidly from the body, mostly unmetabolized in urine [29,30,31,32].

It has been shown that orally administered αCD at a dose of 200 mg/kg body weight (BW) in rats exhibited an absorption rate of 1%. Excretion of intact αCD after oral gavage was carried out via the kidneys. In this context, it is interesting that no intact αCD was found in the feces, which indicates that αCD is completely fermented by the intestinal microbiota, such as resistant starch or other types of fermentable dietary fiber [30]. Intravenous administration of 14C-labelled αCD to Wistar rats indicated half-lives of 21 and 26 min in blood in male and female rats, respectively. Furthermore, it has been shown that αCD is excreted rapidly in urine, and only approximately 1.9% of systemic αCD is eliminated with bile or saliva [30].

Male Wistar rats employed by Kubota et al. (1996) received βCD orally at a dose of 500 mg/kg. Here, βCD reached the maximum plasma concentration within 40 min with an absorption rate of 0.6% [29]. Intravenously administered βCD (25–100 mg/kg) exhibited a half-life of 22–26 min in blood. The urinary excretion of intact βCD was approximately 90% within 10 h [29] or 24 h [32] after intravenous administration. According to these findings, βCD is essentially eliminated via urine without undergoing relevant metabolism in rats [29,32].

The enzyme β-amylase hydrolyses starch from the nonreducing end. Although γCD is resistant to degradation by β-amylase, it is a substrate of salivary and pancreatic α-amylase, whereas α- and βCD are not. The α-amylase hydrolyses α-bonds from large polysaccharides such as starch. γCD is almost completely degraded to malto-triose, maltose, and glucose, similar to the digestion of starch and linear dextrins [5,33,34]. The degradation of γCD through α-amylase starts with a ring-opening reaction, which is the slowest step of the degradation process of γCD. Linear malto-octaose is the result of this ring-opening reaction, and can be further degraded by amylases [34]. It has been assumed that the higher number of glucose molecules in γCD than in α- and βCD could be why γCD can be degraded by α-amylase. The eight glucose monomers of γCD and the higher flexibility in its circular structure result in higher susceptibility to the opening reaction of amylases [34]. Glucose is readily absorbed from the gastrointestinal tract, whereas the absorption of intact γCD has been reported to be very low (0.02%) [31]. Moreover, the formation of inclusion complexes with γCD decreased hydrolysis by α-amylase [35]. Intravenously administered 14C-γCD indicated a half-life of 15–20 min in blood. Excretion kinetics showed that approximately 90% of γCD was excreted in urine within 24 h [31].

## 6. Safety of Cyclodextrins

A high parenteral dose of βCD (200 mg/kg) in rats led to a decreased elimination rate, suggesting that this high dose may be nephrotoxic [32]. The renal toxicity of high parenteral doses of αCD [36] and βCD, respectively, has been reported elsewhere [36,37]. The occurrence of crystals in renal tissue might explain the nephrotoxicity [36,37]. However, a dietary level of αCD up to 20% did not reveal adverse effects or signs of toxicity in studies in rats and beagle dogs [38,39]. In healthy humans, treatment with 6 g αCD per day for 12–14 weeks in a randomized, double-blind clinical trial was well tolerated. Only mild gastrointestinal symptoms occurred and may be side effects related to oral αCD administration [40]. The toxicity of βCD was studied in Sprague-Dawley rats and beagle dogs receiving orally administered βCD for up to 52 weeks. The nontoxic effect level in rats was 654 or 864 mg/kg/day for males or females, respectively, and 1831 or 1967 mg/kg/day for male dogs or female dogs, respectively [41].

No signs of toxicity from γCD have been shown in acute toxicity studies in mice and rats after oral, subcutaneous, intravenous, and intraperitoneal administration [5]. A dietary level of up to 20% γCD has been shown to be well tolerated without toxic side effects on the basis of studies in beagle dogs and Wister rats [5,42]. A dietary level of up to 20% γCD in pregnant female rats did not reveal any fetotoxic, embryotoxic, or teratogenic effects [43]. The oral toxicity of up to 20% γCD in the diet was examined in beagle dogs over a 13-week period. The only treatment-related observed effects were transient diarrhea and cecal enlargement. Both side effects are well-known physiological responses to high doses of orally administered carbohydrates [42]. In humans, a single oral dose of 50 g of carbohydrate from γCD or maltodextrin was administered in a double-masked, randomized, crossover study with 32 healthy adult subjects. The treatments were both well tolerated [44].

Overall, all three parent CDs are “generally recognized as safe” (GRAS) by the U.S. FDA for use as a food additive [45,46,47]. Furthermore, βCD is approved in Europe as a food additive (E459) with an acceptable daily intake (ADI) of 5 mg/kg BW per day [48]. However, the European Medicines Agency (EMA) recommends against the parenteral use of αCD and βCD due to their hepatotoxicity, although there has been one intravenous product containing αCD on the market in Japan [49].

## 7. Cyclodextrin Inclusion Complexes

During complexation, guest molecules and CD molecules come into contact with each other to unite and form a complex [50]. In most cases, one guest molecule forms a complex with one CD molecule, resulting in a 1:1 type of CD complex (Figure 4b) [4,26,51].

In aqueous solutions, the internal cavity of CDs is occupied by water molecules. These water molecules can be substituted by less polar guest molecules. The driving force is the polar–apolar interaction between water and CD, which results in an energetically unfavored environment. Complex formation takes place through substitution with an appropriate guest molecule or, more commonly, some lipophilic moiety of the molecule, which is less polar than water molecules [4,8]. This process reduces the total energy of the system and causes a change in enthalpy, resulting in enhanced stability of the complex [21], while no covalent bonds are formed or broken [4]. Therefore, water is almost necessary in the formation of complexes [50]. During the complexation process, negative values of free energy changes by the Gibbs equation have been shown for ursolic acid (UA) and oleanolic acid (OA) with βCD and γCD, respectively, indicating that complexation was a spontaneous process [24,26].

Various methods have been applied to prepare CD complexes: The solution method, coprecipitation method, neutralization method, slurry method, kneading method, and grinding method [4,50]. Factors such as temperature, amount of water, mixing time, and drying conditions have to be adapted for each guest molecule and CD complex [50]. For example, the triterpenoids UA, OA, betulinic acid (BA), and betulin (Bet) formed complexes with CD when using the kneading method [52,53,54]. Both BA and Bet were encapsulated in a γCD derivative with a molar ratio of 1:1. An equal quantity of ethanol and water (1:1) was used as the solvent mixture. All materials were continuously kneaded together for several minutes until the majority of the solvent mixture had been evaporated. The resulting mixture was dried at room temperature for 24 h and then in an oven at 105 °C for 7 h. Then, the final product was pulverized and sieved [52,54].

**Figure 5:**
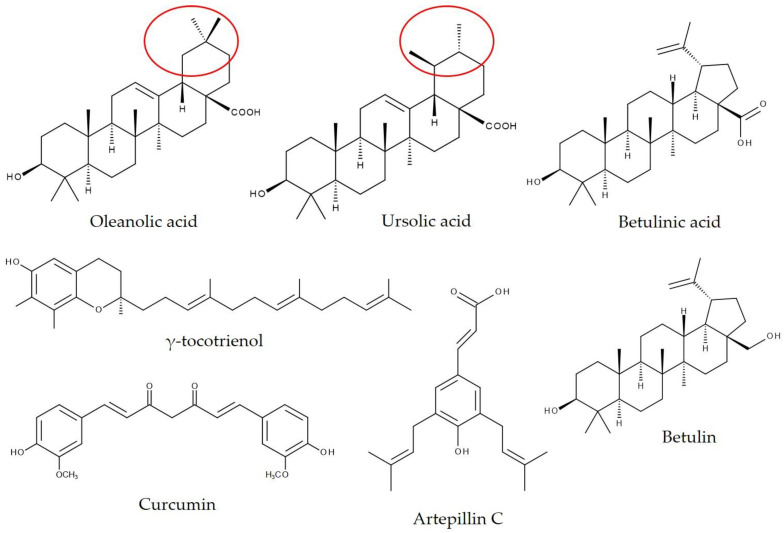
Chemical structures of oleanolic acid, ursolic acid, betulinic acid, γ-tocotrienol, curcumin, artepillin C, and betulin. Oleanolic acid and ursolic acid differ in the location of one methyl group on ring E [26], highlighted by the red circles.

The host-guest interaction of OA and UA with βCD was studied by Huang et al. [26]. They found that the kneading method resulted in products with higher drug loading, but limited improvement in solubility. Therefore, the authors chose the stirring method because the stirred products showed better inclusion and were more easily obtained [26]. Furthermore, it has been shown that both the stability constant (K) and the complexation efficiency (CE) of UA were higher than those of OA. Because OA and UA have a very similar chemical structure except for the location of one methyl group on ring E (Figure 5), it has been assumed that ring E of both triterpenoids was encapsulated in the CD cavity [26]. For further information about the mechanism behind complexation, the hydrogen bonding parameters were analyzed. Two intermolecular hydrogen bonds were revealed between OA or UA and βCD. The hydrogen bonds were formed between the H atom on the carbonyl group of UA (OA) and the O atom in βCD and between the O atom in the carbonyl group of UA (OA) and the H atom bonding to the C atom in βCD [26,26]. The most stable complex structure was reported to have the carboxyl group oriented to the center of the CD cavity [24,26].

Another studied complex is caffeic acid phenethyl ester (CAPE)-γCD. The structure of CAPE, a key anticancer phenolic compound in New Zealand propolis, as well as the complex structure of CAPE-γCD, is shown Figure 4c [23]. It is suggested that the caffeic acid moiety is encapsulated in the CD cavity.

## 8. Application of Cyclodextrins

In 2017, approximately 70% of global CD production consisted of βCD, while the shares of αCD and γCD were approximately 15% and 5%, respectively. However, due to its favorable toxicological profile, γCD, which was initially less produced, is becoming increasingly attractive as a pharmaceutical excipient [5]. In Japan, native CDs are regarded as natural products, resulting in usage without many restrictions both in medicines and in foods [11]. CDs have been used in food and pharmaceutical products for many years, largely to form inclusion complexes with problematic drugs to enhance their solubility [55]. In addition, previous studies have shown that CDs are useful as an antibacterial food packaging material when forming inclusion complexes with natural antimicrobial agents such as thymol and carvacrol [56,57,58,59,60,61,62,63,64,65]. This embedment of an antibacterial agent into food packaging material is one strategy to inhibit bacterial growth. Mostly, βCD was successfully employed as a host molecule. However, Aytac et al. [56] tested thymol-γCD inclusion complex-encapsulated electrospun zein nanofibrous webs (zein-THY-γCD-NF) as a potential food packaging material for meat samples. The electrospun nanofibers were prepared from a solution of THY-γCD complex-incorporated zein. The antibacterial activity of zein-THY-γCD-NF against *Escherichia coli* and *Staphylococcus aureus* was higher than that of zein-THY-NF without γCD. Therefore, the webs are of interest for application as antibacterial food packaging materials [56].

As reported by Fenyvesi, Vikmon, and Szente [27], the food application of CDs started in the 1970s. In particular, γCD has received much attention in oral bioavailability experiments and is regarded as a promising nutrition delivery system [22]. An overview of bioavailability and bioactivity of inclusion complexes of tocotrienols, pentacyclic triterpenoids, propolis and curcumin with γCD is given in Table 1. γCD can stabilize diverse food factors, such as flavors, sensitive colors, fat-soluble vitamins, polyunsaturated fatty acids, and emulsions of fats and oils. Such inclusion complexes are useful for the formulation of meal replacements in powder form and of dietary supplements [33]. However, one must keep in mind that hydrophilic CDs can act only as carrier materials for lipophilic guest molecules in oral administration by helping to transport them safely through an aqueous medium to the absorption surface of the gastrointestinal tract [66], thereby increasing their stability only until they permeate biological membranes of, for example, the intestinal epithelium.

Since CDs are poorly absorbed through biological membranes due to their relatively high molecular weight and large number of hydrogen donors and acceptors [4], it is not surprising that they usually do not increase the permeability of hydrophilic guest molecules through lipophilic biological membranes [4]. However, CDs can stabilize lipophilic guest molecules until they reach the unstirred water layer adjacent to the membrane surface of the biological barrier [4]. Generally, only a free form of the lipophilic guest molecule is able to penetrate the lipophilic biological membrane. Binding within the CD cavity is a reversible process, and the free and complexed forms of the drug are in equilibrium in solution [66]. This equilibrium is compound-specific. Accordingly, CDs cannot increase the bioavailability of every kind of molecule. For example, Class II drugs (Biopharmaceutics Classification System categories) have poor aqueous solubility, but show good membrane permeability. Here, complexation with CDs can enhance oral bioavailability by increasing diffusion to the mucosal surface. It has been reported that in this case, the most important factor inhibiting bioavailability is the low aqueous solubility of the drugs, which hinders their dissociation from the molecule as well as their permeation through the water layer adjacent to the membrane surface [4]. The bioavailability of Class I drugs cannot be improved by CDs. They are defined as highly water soluble and highly membrane permeable, and therefore have good bioavailability after oral administration [4]. Hence, such compounds are generally not encapsulated into CDs to enhance their bioavailability. A detailed review of drug absorption after oral administration and other application routes is given by Loftsson et al. [4] and Uekama et al. [66].

Regarding the use of CDs in pharmaceutical products to form complexes with lipophilic drugs, parenteral application also matters. It should be noted that parenteral administration is mainly suitable for βCD derivatives such as HPβCD. αCD and βCD are not recommended because of their reported nephrotoxicity. The low aqueous solubility of βCD is also not an advantage for its use as a parenteral drug [49]. Similarly, γCD, which forms visible aggregates in aqueous solutions, is not suitable for parenteral formulations [21]. Furthermore, CDs have a short half-life in systemic circulation and a high excretion rate in urine, as described above. In the short time in systemic circulation, the complex may not reach its target organ. Otherwise, the complex may dissociate before it reaches the drug target organ or tissue [66].

### 8.1. Inclusion of Tocotrienol in γ-Cyclodextrin Increased Its Bioavailability and Bioactivity

A study in male Wistar rats examined the effects of different tocotrienol emulsions (13.9 mg tocotrienol) with or without γCD on γ-tocotrienol (for chemical structure, see Figure 5) concentrations in plasma and tissues. At 3 h after the oral administration of tocotrienol, tocotrienol with γCD, or tocotrienol/γCD-complex, the complex of tocotrienol with γCD led to elevated plasma and tissue concentrations of γ-tocotrienol compared to the simultaneous administration of tocotrienol and γCD. The authors suggested that complexation causes improved solubility and stability of tocotrienol in the gastrointestinal tract, thereby enhancing its intestinal absorption. Pretreatment with the detergent Triton and subsequent oral gavage of the different emulsions verified this suggestion in the laboratory rodents. The rats that were given the Triton complex exhibited higher plasma γ-tocotrienol concentrations than the animals in the other two groups. The study also clarified that the tissue accumulation of tocotrienol is less regulated than plasma concentration [67]. It has also been demonstrated that the inclusion of γ-tocotrienol in γCD significantly improved the oral bioavailability and physiological activity of γ-tocotrienol in young C57BL/6 mice. The mice of the control group received 2.79 mg of a γ-tocotrienol-rich fraction extracted from rice bran, while the complex group obtained an equivalent dose of the γ-tocotrienol-rich fraction included in γCD. Complexation led to a 1.4-fold increase in the area under the curve of γ-tocotrienol plasma concentration compared to the γ-tocotrienol-rich fraction only [22]. In addition, the complexation of tocotrienols with γCD resulted in a prolonged life span in the nematode *Caenorhabditis elegans* compared to worms receiving pure γCD or pure tocotrienols. Initially, it was observed that the nematodes absorbed a fluorescent dye, which was encapsulated in γCD for this assay, from the gastrointestinal tract and accumulated the dye in the cytoplasm of the intestinal cells. After testing the ingestion of γCD inclusion compounds, the oral administration of tocotrienols in γCDs was analyzed. Accordingly, the authors suggested that γCD is an excellent vehicle for the oral gavage of hydrophobic substances such as tocotrienol [68]. Schloesser et al. [69] evaluated the effect of a dietary tocotrienol-γCD complex on an ageing brain phenotype in mice. Male middle-aged C57BL/6J mice received a high-fat, high-sugar Western-type diet with or without (control) tocotrienol-γCD complex for up to 24 weeks. The tocotrienol content was 100 mg/kg diet. Examination of the brain revealed significantly increased mitochondrial membrane potential and ATP levels in the complex mice compared to the controls [69]. Unfortunately, the authors did not include a control group with tocotrienols only.

In summary, tocotrienol exhibits improved bioavailability through enhanced solubility and stability in the gastrointestinal tract, and therefore enhanced bioactivity in various laboratory model organisms, attributable to encapsulation into CDs.

### 8.2. Pentacyclic Triterpenoids Encapsulated in γ-Cyclodextrin

Pentacyclic triterpenoids such as UA have been reported to act as chemopreventive agents [77,78]. However, due to their low solubility and limited oral bioavailability [79], new approaches, such as encapsulation into CDs, have been introduced to enhance bioavailability and bioactivity. Several cell culture studies have demonstrated that complex formulations of triterpenoids and CDs could improve the anticancer activity of triterpenoids [24,51,53,80]. This seems somewhat questionable, especially when looking at studies where a significant effect was shown in vivo, but not in vitro [52,54]. However, a possible explanation for the improved in vitro anticancer activity might be that CDs enhance the cellular uptake of drugs [24] by simply increasing their solubility in aqueous media.

Soica et al. [53] found synergistic antiproliferative activity of a mixture of OA and UA (for chemical structure, see Figure 5) encapsulated in 2-hydroxypropyl-γ-cyclodextrin (HPγCD) when applied in a chemically (7,12-dimethylbenz(a)-anthracene/12-O-tetradecanoyl-phorbol-13-acetate) induced and UV-induced murine skin cancer model. SKH1 female mice were treated 30 min before the application of carcinogens with either 200 µL of a 2% aqueous solution of OA-HPγCD, UA-HPγCD, or OA/UA-HPγCD. The delta of transepidermal water loss (TWL) was in the control group at approximately 25 units/six weeks. The mice treated with UA-HPγCD and OA/UA-HPγCD showed practically no modification in TWL. The application of OA/UA-HPγCD led to the greatest increase in skin pH. The evaluation of erythema, an important skin parameter involved in the assessment of drug or chemical irritative potential, indicated a change of more than 230 units after six weeks for the control group. However, only a small difference was documented for the mice who received UA-HPγCD or OA/UA-HPγCD. Similar results were reported for water loss from the stratum corneum [53].

Betulin (Bet) and betulinic acid (BA) (Figure 5) were encapsulated in octakis-[6-deoxy-6-(2-sulfanyl ethanesulfonate)]-γCD (OγCD), a γCD derivative, to analyze their antitumor activity in a B164A5/C57BL/6J mouse melanoma model [52,54]. At 2 days after the inoculation of B164A5 cells in mice, the Bet-OγCD complex was given intraperitoneally daily for 14 days at a concentration of 20 mg Bet/BW [54]. In BA-OγCD-treated mice, the complex was administered intraperitoneally 1 day after cell inoculation at a dose of 100 mg/kg daily for 3 weeks [52]. Both treatments significantly inhibited tumor growth in mice compared to that in untreated laboratory animals [52,54]. BA-OγCD administration further led to decreased melanin, erythema, and TWL levels compared to control mice [52]. Natural sources of UA (homogenate apple peel, HAP, and micronized apple peel, MAP) were compared with encapsulated UA in βCD and γCD, respectively, regarding their impact on liver regeneration. Therefore, male Wistar rats received 20 mg UA daily for 7 days via intragastric gavage. On day 6, a partial hepatectomy (70%) was conducted. UA-treated rats showed increased liver regeneration in comparison to untreated mice. In particular, the UA complex with γCD exhibited good results. Plasma levels and expression of hepatocyte growth factor in the liver were significantly increased in the high-fat diet-UA-γCD group compared to the control group [70].

However, in a study that used dietary Kuding tea extract as a UA source (KTE, 7.12%) encapsulated in 12.88% γCD (comprising 150 mg UA/kg BW), young male C57BL/6 mice had increased liver weight and hepatic fat accumulation compared to control mice, which received only a high-fat, high-fructose, Western-type diet for 6 weeks. In addition, the mice from the complex group showed increased hepatic peroxisome proliferator activated receptor gamma (Pparγ) and CD36 molecule (Cd36) mRNA levels as well as elevated plasma cholesterol levels and increased cytochrome P450, family 7, subfamily a, polypeptide 1 (CYP7A1) mRNA, and protein levels. Analyzing the enzymes of hepatic xenobiotic metabolism showed that there was a substantial elevation of cytochrome P450, family 3, subfamily a (CYP3A) and glutathione S-transferase, alpha 1 (GSTA1) mRNA, and protein levels in KTE-γCD mice. Pure UA-treated mice exhibited a moderate elevation of CYP3A and GSTA1. In line with the assumption that CDs can mask the taste of guest molecules [3], the study demonstrated that mice receiving a diet with bitter-tasting KTE alone completely refused this diet, while the diet supplemented with KTE-γCD was eaten by mice [71]. This study may show the other side of the coin of some inclusion complex formulations. By encapsulating bioactive compounds, one may not only increase their positive biological effects, but also promote some adverse effects, such as hepatotoxicity.

### 8.3. Propolis, Propolis Extract, or Phytochemicals Isolated from Propolis Encapsulated in γ-Cyclodextrin

Brazilian green propolis supercritical extract (GPSE) has been reported to be rich in artepillin C (3,5-diprenyl-4 hydroxycinnamic acid, Figure 5) and to stimulate immune function. Accordingly, dietary GPSE encapsulated in γCD (2.3 g/kg GPSE-γCD) showed anti-inflammatory properties in female C57BL/6 mice after 10 weeks of supplementation. Hepatic gene expression of tumor necrosis factor alpha (TNFα) and serum amyloid P (Sap) was significantly decreased in GPSE-γCD-fed mice compared to the control group and the γCD-vehicle group, respectively [72]. Moreover, there are several reports on propolis in combination with γCD and its anticancer and antimetastatic activities [73,74,75]. Caffeic acid phenethyl ester (CAPE, Figure 4), a key phenolic component in New Zealand propolis, showed antitumor and antimetastatic potency in female Balb/c nude mice, and the complexation of CAPE with γCD enhanced its activities. γCD per se did not exhibit any bioactivity. The human fibrosarcoma cell line HT1080 was injected subcutaneously into the abdomen of mice and into the tail vein. Starting one day after injection, treatment with CAPE or CAPE-γCD was performed on every alternate day. Mice received either intraperitoneal or oral administration of CAPE for up to 30 days. For both routes of administration, CAPE showed significant tumor suppression and reduction in lung metastasis. CAPE-γCD-treated mice exhibited increased antitumor and antimetastatic activities compared to pure CAPE [73]. According to the findings by Wadhwa et al. [73], in an in vitro cell viability assay, CAPE-γCD revealed higher cytotoxicity in A549 and HT1080 cells than CAPE alone [23]. In addition, the comparison of CAPE and CAPE-γCD solubility in 1.0% taurocholic acid solution showed that complexation with γCD increased the solubility of CAPE in a mimicked intestinal environment [23], possibly resulting in higher tumor suppression activity, as reported in an earlier study [73]. In this context, it is interesting that CAPE alone is vulnerable to digestive enzymes such as secreted esterases. However, when encapsulated in γCD, CAPE is protected and shows enhanced activity [73]. The antitumor properties of GPSE from Brazilian green propolis were also examined. GPSE, containing 9.6% artepillin C and GPSE-γCD, containing 3% artepillin C, was orally administered to BALB/c nude mice beginning 1 day after subcutaneous tumor xenografts for 3 weeks. The authors observed decreased tumor growth in mice fed GPSE and GPSE-γCD compared to untreated mice. The data on GPSE alone and with γCD regarding tumor growth suggested that complexation results in a more effective molecule [74].

Cho et al. [75] reported on C57BL/6J-ApcMin/+/J mice that received either a control diet for a lean model or a high-fat diet for an obese model supplemented with γCD and propolis (containing γCD), for 8 weeks. The chosen mouse model tends to develop intestinal cancer with adenomas. In the lean model, γCD and propolis-treated mice showed decreased neoplastic progression. Supplementation with propolis led to a further decrease in the number of adenomas compared to the control diet [75]. However, since γCD also affects intestinal tumor development, it is not exactly clear what part propolis plays in this effect.

### 8.4. Increased Bioavailability of Curcumin by Complexation with γ-Cyclodextrin in Humans

Curcumin (Figure 5) is the main bioactive hydrophobic polyphenolic compound from the so-called curcuminoids provided by turmeric, a member of the ginger family [76,81]. Previous studies have shown that curcumin possesses various pharmacological activities, such as antioxidant, anti-inflammatory, and neuroprotective properties [82]. However, the metabolism of curcumin, particularly in the intestine and liver, determines its bioavailability and bioactivity [81]. Due to the low water solubility and poor intestinal absorption of curcumin, different formulations have been developed to enhance its bioavailability [76]. In this context, Purpura et al. [76] analyzed the pharmacokinetics of a standardized unformulated curcumin extract (StdC), a γCD-curcumin formulation (C-γCD), a curcumin phytosome formulation, and a curcumin formulation with essential oils of turmeric extracted from the rhizome in a double-blind, crossover study in 12 healthy human subjects. The curcumin content in C-γCD was determined to be 348 mg. In addition, the complex formulation contained demethoxycurcumin and bisdemethoxycurcumin, resulting in a total curcuminoid content of 371 mg. Complexation with γCD significantly increased the bioavailability of curcumin (85-fold) and total curcuminoids (39-fold) compared to StdC. Plasma levels of curcuminoids were measured by HPLC-MS/MS analysis up to 12 h after the intake of hard gel capsules of each of the different curcumin formulations. The peak concentration of curcumin from C-γCD in plasma was 73 ng/mL, achieved after 1 h. However, the peak curcumin concentration after StdC intake was 0.5 ng/mL after 12 h [76].

### 8.5. Metabolic Activity of Empty γ-Cyclodextrin

As mentioned above, the propolis-γCD study by Cho et al. [75] also revealed an unexpected beneficial effect of γCD supplementation per se. C57BL/6J-ApcMin/+/J mice were fed either a control diet for a lean model or a high-fat diet for an obese model supplemented with γCD and propolis (containing γCD) for 8 weeks. Both control groups treated with γCD showed modulation of intestinal tumor development. In the obese state, dietary γCD led to the lowest number and size of adenomas and concurrent to the highest percentage of lesions with low grades of dysplasia in mice. Interestingly, the laboratory animals supplemented with propolis (containing γCD) did not show this reduction in neoplastic burden, indicating that propolis counteracted these effects. It has been assumed that the effect of dietary γCD resulted from enhanced levels of apoptosis in intestinal tissue, achieved by butyrate derived from γCD metabolism [75].

Another interesting biological effect of empty γCD was provided by Asp et al. [44]. Thirty-two healthy adult subjects received 50 g of carbohydrate from either γCD or maltodextrin during a double-blind, randomized crossover study. Plasma glucose and serum insulin levels were reported for up to 180 min postprandial. Intake of γCD led to a moderate and gradual increase in both glycaemia parameters. The area under the curve (AUC) of plasma glucose was 45% reduced compared to maltodextrin. Consequently, the AUC of serum insulin was reduced by 49% by γCD compared to maltodextrin. Monitored breath hydrogen excretion showed no differences between the two carbohydrate sources, indicating that γCD is fully hydrolyzed in the small intestine. In comparison with rapidly digested maltodextrin, γCD is assumed to be a slowly digested carbohydrate, resulting in reduced postprandial glycaemia [44]. In line with this assumption, it has been reported that dietary γCD leads to increased endurance in C57BL/6 mice, probably due to a prolonged supply of glucose during exercise. The laboratory animals received a control diet or a diet supplemented with 12.88% γCD for 6 weeks. Voluntary activity was monitored via the wheel running behavior of mice. The γCD-treated animals covered a significantly larger distance per night and were active significantly longer during the night than the control mice. Furthermore, adding γCD to the diet led to a significantly better performance of mice in the inverted screen test, suggesting that these animals showed enhanced muscle strength. It was also observed that dietary γCD had some slight antiglycemic effects [83].

The existing literature data demonstrate that the γCD derivative HPγCD may act as a therapeutic approach for Niemann-Pick type C disease (NPC) [84,85,86,87,88], which is a fatal neurodegenerative disorder characterized by a massive accumulation of free cholesterol and other lipids in late endosomes and lysosomes. In addition, NPC1-deficient cells showed defects in autophagy [89,90]. In line with this assumption, it has been reported that HPγCD alleviates cholesterol accumulation in NPC1 patient-derived fibroblasts by modulating lysosomal dynamics and functions as well as enhancing autophagic activity [84,85,87,91]. The cholesterol-binding capacity of HPγCD is still lower than that of HPβCD [86,91], but the same applies for its ototoxicity [86]. Therefore, HPγCD might be a more promising drug candidate than HPβCD for the treatment of NPC. In particular, HPγCD alleviates lysosomal cholesterol accumulation in NPC1-deficient cells to the same degree as HPβCD [84], or even more effectively [87]. Moreover, liver dysfunction and cholesterol accumulation were enhanced by the subcutaneous injection of HPγCD in NPC model mice [87]. Recent molecular research has indicated that the effects of HPγCD are partly mediated by transcription factor EB, which is a regulator of lysosomal functions and autophagy [84]. However, the underlying mechanism of action remains to be clarified. Hoque et al. [88] showed that both HPβCD and HPγCD (but not HPαCD) reduced cholesterol and sphingolipid accumulation solely in Npc1-null Chinese hamster ovary cells, but not in the corresponding control wild-type cells.

## 9. Conclusions and Outlook

Increasing the stability, bioavailability, and bioactivity of natural compounds and plant bioactives is an important issue in food science and pharmaceutics. As reviewed here, this goal can be achieved by encapsulating these often lipophilic molecules in γCD. A growing number of studies indicate that such CD complexes often lead to enhanced biological/pharmacological efficiency, increased stability, and better taste and odor.

However, one should consider that improving the bioavailability of dietary plant bioactives may be accompanied by some risks, and a critical evaluation as well as a risk assessment is needed. Encapsulation enables the intake of higher dosages of natural compounds and plant bioactives. As shown for KTE encapsulated in γCD, high dietary supplementation can lead to an increased hepatic phase I and phase II response [71]. Hence, improving the bioavailability of plant bioactives that undergo biotransformation can cause hepatotoxicity due to exceeding the toxicity threshold or can lead to herb-drug interactions, thereby affecting medical treatment [92]. In this regard, it is notable that the number of liver injury cases in the United States associated with dietary supplementation of herbal sources is actually increasing [93].

Similarly, the complexation of natural products such as the bee product propolis could also increase the bioavailability of potential allergens. Propolis allergies per se were more of an occupational disease affecting beekeepers in the past. However, since propolis is frequently included in dietary supplements, more and more, an increasing number of people are affected by this allergy [94].

Moreover, by complexing herbal extracts into CDs, one may increase the bioavailability of undesirable substances such as pesticides or contaminants. The selection and quality of the raw material is therefore of particular importance. It should also be considered that CDs mask the bitter taste and odor of their complexation agents, as reported for KTE. A bitter taste often results in food aversion by animals, thereby protecting them against the consumption of toxic compounds, although not all bitter compounds are toxic [95].

CDs have a long history as host molecules in inclusion complexes and were long presumed to be inert molecules. However, only recently has it become evident that CDs are not just carrier molecules, but also undergo digestion and fermentation in the digestive tract. In particular, the first studies on γCD supplementation revealed promising effects on energy metabolism. Therefore, the impact of empty CDs on metabolism and their possible nutritional or pharmaceutical applications need additional investigation.

## Figures and Tables

**Table 1:** Bioavailability and bioactivity of inclusion complexes of tocotrienols, pentacyclic triterpenoids, propolis, and curcumin with γ-cyclodextrin.

Substances and Cyclodextrins	Molar Ratio	Model Organism	Administration	Dose	Outcome	Authors
Tocotrienol (T3) and γ-cyclodextrin (γCD)		Wistar rats n = 5–8	Oral	13.9 mg T3	Improved solubility and stability in the GIT; enhanced intestinal absorption of T3; increased plasma and tissue concentration of T3	Ikeda et al. 2014 [67]
T3-rich fraction (TRF) from rice bran and γCD		C57BL/6 mice n = 3 (bioavailability) n = 12–15 (bioactivity)	Oral	2.79 mg TRF	1.4-fold increase of AUC of T3 plasma concentration; improved bioavailability and physiological activity	Miyoshi et al. 2011 [22]
T3 and γCD		*Caenorhabditis elegans* n = 30 worms per group/duplicates, performed two or three times	Oral	26, 86, and 259 µg T3	Prolonged lifespan	Kashima et al. 2012 [68]
T3 and γCD		C57BL/6 mice n = 6–8	Oral	100 mg T3/kg diet	Increased mitochondrial membrane potential and ATP levels in aging brain	Schloesser et al. 2015 [69]
Ursolic acid (UA) & oleanolic acid (OA) & 2-hydroxypropyl-γCD (HP-γCD)		SKH1 mice n = 6	Dermal	200 µL 2% aqueous solution	No modification in TWL; increased skin-pH; small change of erythema; small difference in *stratum corneum* moisture content; anti-tumor activity	Soica et al. 2014 [53]
Betulin (Bet) and octakis-γCD	1:1	C57BL/6 mice n = 5	Subcutaneous injection	20 mg Bet/BW	Decreased tumor volume; decreased tumor weight	Soica et al. 2012 [54]
Betulinic acid (BA) and octakis-γCD	1:1	C57BL/6 mice n = 10	Subcutaneous injection	100 mg BA/kg	Decreased tumor volume; decreased tumor weight; decreased melanin, erythema, and TWL levels	Soica et al. 2014 [52]
UA and γCD		Wistar rats n = 6	Intragastric	20 mg UA	Increased liver regeneration; increased hepatocyte growth factor liver expression and plasma levels	Žaloudková et al. 2020 [70]
Kuding tea extract (KTE) and γCD	1:2	C57BL/6 mice n = 8–10	Oral	7.12% KTE (comprising 0.15% UA)	Increased liver weight; elevated hepatic fat accumulation; increased hepatic PPARγ, CD36, CYP7A1, CYP3A, and GSTA1 levels; increased plasma cholesterol level	Wüpper et al. 2020 [71]
Brazilian green propolis supercritical extract (GPSE) and γCD		C57BL/6 mice n = 10	Oral	2.3 g/kg GPSE-γCD (providing 200 mg artepillin C/kg diet)	Decreased hepatic TNFα and Sap mRNA level; anti-inflammatory properties	Rimbach et al. 2017 [72]
Caffeic acid phenethyl ester (CAPE) and γCD	1:1	Balb/c nude mice n = 9	Oral or intraperitoneal		Decreased tumor volume; anti-metastatic properties	Wadhwa et al. 2016 [73]
CAPE and γCD		A549 and HT1080 cells			Higher cytotoxicity; higher solubility in a mimicked intestinal environment	Ishida et al. 2018 [23]
GPSE and γCD	1:1	BALB/c nude mice n = 3	Oral	GPSE-γCD (containing 3% artepillin C)	Decreased tumor growth	Bhargava et al. 2018 [74]
Propolis and γCD		C57BL/6J-*ApcMin*/+/J mice n = 5–6	Oral	8% propolis-γCD (comprising 6% γCD) and pure γCD	Decreased tumor development; decreased number of adenomas	Cho et al. 2016 [75]
Curcuminoids and γCD		Humans n = 12	Oral	371.4 mg total curcuminoids (containing 346 mg curcumin)	Improved solubility and stability in the GIT; enhanced intestinal absorption of T3 increased plasma and tissue concentration of T3	Purpura et al. 2018 [76]

AUC, area under the curve; BA, betulinic acid; Bet, betulin; CAPE, caffeic acid phenethyl ester; CD36, CD36 molecule; CYP3A, cytochrome P450, family 3, subfamily a; CYP7A1, cytochrome P450, family 7, subfamily a, polypeptide 1; GIT, gastrointestinal tract; GPSE, green propolis supercritical extract; GSTA1, glutathione S-transferase, alpha 1; HPγCD; 2-hydroxypropyl-γCD; KTE, Kuding tea extract; OA, oleanolic acid; PPARγ, hepatic peroxisome proliferator activated receptor gamma; Sap, serum amyloid P; T3, tocotrienol; TNFα, tumour necrosis factor alpha; TRF, T3-rich fraction; TWL, transepidermal water loss; UA, ursolic acid; γCD, gamma-cyclodextrin.

## Data Availability

Data sharing not applicable.
